# PM_2.5_ Exposure Induces Glomerular Hyperfiltration in Mice in a Gender-Dependent Manner

**DOI:** 10.3390/toxics12120878

**Published:** 2024-12-01

**Authors:** Hao Wang, Li Ma, Yuqiong Guo, Lingyu Ren, Guangke Li, Nan Sang

**Affiliations:** Shanxi Key Laboratory of Coal-Based Emerging Pollutant Identification and Risk Control, Research Center of Environment and Health, College of Environment and Resource, Shanxi University, Taiyuan 030006, China; wh1034824679@163.com (H.W.); ml384000330@163.com (L.M.); 18635827695@163.com (Y.G.); renlingyu9619@163.com (L.R.); liguangke@sxu.edu.cn (G.L.)

**Keywords:** PM_2.5_, renal hyperfiltration, renin–angiotensin system, kallikrein–kinin system, tubular reabsorption, tubuloglomerular feedback

## Abstract

As one of the most common air pollutants, fine particulate matter (PM_2.5_) increases the risk of diseases in various systems, including the urinary system. In the present study, we exposed male and female C57BL/6J mice to PM_2.5_ for 8 weeks. Examination of renal function indices, including creatinine (CRE), blood urea nitrogen (BUN), uric acid (UA), and urinary microalbumin, indicated that the kidneys of female mice, not male mice, underwent early renal injury, exhibiting glomerular hyperfiltration. Meanwhile, pathological staining showed that the kidneys of female mice exhibited enlarged glomerulus that filled the entire Bowman’s capsule in the female mice. Afterward, we explored the potential causes and mechanisms of glomerular hyperfiltration. Variations in mRNA levels of key genes involved in the renin–angiotensin system (RAS) and kallikrein–kinin system (KKS) demonstrated that PM_2.5_ led to elevated glomerular capillary hydrostatic pressure in female mice by disturbing the balance between the RAS and KKS, which in turn increased the glomerular filtration rate (GFR). In addition, we found that PM_2.5_ increased blood glucose levels in the females, which enhanced tubular reabsorption of glucose, attenuated macular dense sensory signaling, induced renal hypoxia, and affected adenosine triphosphate (ATP) synthesis, thus attenuating tubuloglomerular feedback (TGF)-induced afferent arteriolar constriction and leading to glomerular hyperfiltration. In conclusion, this study indicated that PM_2.5_ induced glomerular hyperfiltration in female mice by affecting RAS/KKS imbalances, as well as the regulation of TGF; innovatively unveiled the association between PM_2.5_ subchronic exposure and early kidney injury and its gender dependence; enriched the toxicological evidence of PM_2.5_ and confirmed the importance of reducing ambient PM_2.5_ concentrations.

## 1. Introduction

Ambient fine particulate matter (PM_2.5_)—characterized by a small particle size (all abbreviations in Table 1); a large relative surface area; and a complex composition containing heavy metals, polycyclic aromatic hydrocarbons (PAHs), carbonaceous particles (CP), and other organic compounds— is a leading risk factor in global health, and the health problems it poses have aroused widespread public concern [1]. While some countries, such as China, have seen significant reductions in PM_2.5_ levels in the past few years, very few countries and regions meet the World Health Organization’s Air Quality Guidelines for PM_2.5_ [2]. PM_2.5_ can enter the lungs via the respiratory tract and further enter the bloodstream, leading to various adverse health effects. Epidemiological studies indicate that PM_2.5_ exposure can increase global deaths and disability-adjusted life years (DALYs) [3], as well as the prevalence of anemia, acute respiratory infections, and diseases of other systems [4]. Toxicological studies have suggested that PM_2.5_ not only causes respiratory, cardiovascular, and neurological diseases but also accumulates in distal organs, such as the liver and kidneys [5,6]. In recent years, kidney disease has become a hidden epidemic worldwide. Studies have demonstrated that air pollution, including PM_2.5_, contributes to decreased kidney function and accelerates the development of renal diseases [7,8,9]. On the one hand, PM_2.5_ exposure causes renal impairment by directly inducing oxidative stress, inflammation, cytotoxicity, and angiotensin mediators [2,10,11]. Inflammation is one of the most common mechanisms by which PM_2.5_ produces nephrotoxicity. For example, PM_2.5_ can cause kidney injury through NLRP3-mediated inflammatory activation of macrophages with the activation of the IL-6/STAT3 pathway [12,13]. In addition, PM_2.5_ induces abnormal renal sodium excretion mediated by renal D1 receptors [14]. On the other hand, PM_2.5_ can also indirectly aggravate kidney damage along with the increasing prevalence of obesity, diabetes, hypertension, and genetic factors [15,16]. For example, blood glucose abnormalities can cause changes in the Rho/Rock signaling pathway and the Notch3-mediated mTOR signaling pathway, resulting in kidney injury [17,18]. Currently, studies on the nephrotoxicity of PM_2.5_ mainly focus on acute and long-term exposure, and studies on subacute and subchronic exposure are lacking. Furthermore, PM_2.5_ exhibits strong seasonality and geography. As a typical coal-fired city in northern China, Taiyuan often experiences hazy weather conditions during the winter heating period; it is of great interest to explore the effects of airborne PM_2.5_ in this region on renal function [19]. Furthermore, gender difference is an important factor in disease risk assessment, but few studies have reported on the gender differences in the effects of PM_2.5_ on renal injury in adult mice.

**Figure 1:**
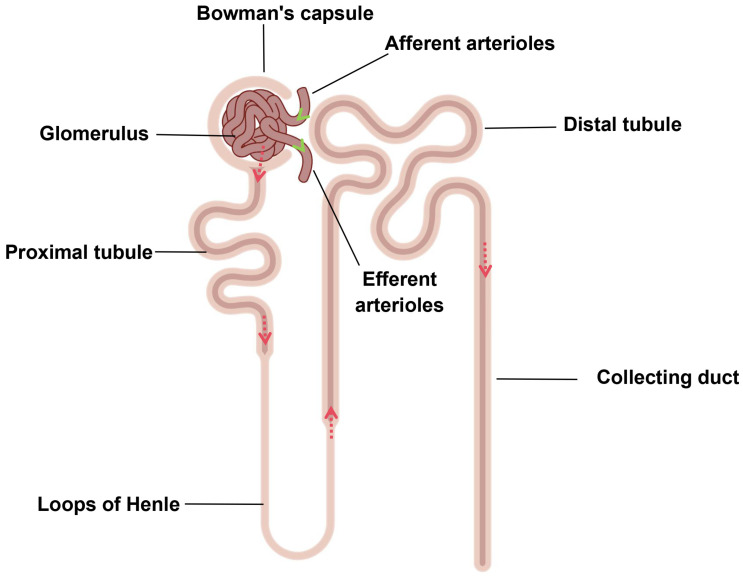
The composition and structure of the renal nephron ( Red arrow represents the process of urine formation and green arrow represents the direction of blood flow. The elements were derived from Figdraw).

The kidney is an important urinary organ with endocrine functions, responsible for filtering blood plasma, excreting metabolic waste, reabsorbing nutrients, and regulating blood pressure to ensure a stable internal environment and normal metabolism [20]. The functional unit of the kidney is the nephron, composed of the renal corpuscle and tubules. The renal corpuscle consists of the Bowman’s capsule and glomerulus, which is formed by capillaries branching off from afferent arterioles, and the branches of each capillary eventually converge to form the efferent arteriole. The tubule consists of the proximal tubule, the loops of Henle, and the distal tubule [21]. Blood enters the kidney via the renal artery and then passes through the afferent arterioles to the glomerulus. Driven by the effective filtration pressure, all components of the blood, except for macromolecules such as proteins and blood cells, travel through the glomerular filtration membrane to enter the Bowman’s capsule and are discharged into the tubules (the proximal tubule, loop of Henle, and distal tubule). After reabsorption, urine is formed (Figure 1) [22]. Kidney injury is often accompanied by changes in the glomerular filtration rate (GFR) as well as alterations in tubular reabsorption function [23,24]. Numerous studies have shown that PM_2.5_ affects the body’s metabolism, causing the development of hyperglycemia, hyperlipidemia, and hypertension, all of which influence the kidneys as the disease progresses.

In this study, we exposed male and female C57BL/6J mice to PM_2.5_ for 8 weeks to investigate its nephrotoxicity in different genders and the underlying mechanisms, thus enriching the toxicological evidence on PM_2.5_ and aiding in the adoption of relevant regulations to reduce PM_2.5_ pollution and improve human health.

## 2. Materials and Methods

### 2.1. Collection and Physicochemical Properties of PM_2.5_

PM_2.5_ was collected in Taiyuan, Shanxi province, China, using quartz filters (Φ90 mm, Munktell, Sweden) and a KC-1000 middle-volume air sampler (Laoshan Electronic Instrument, Qingdao, China) at a flow rate of 100 L/min (22 h/day) from November 2018 to March 2019. The extraction, storage, and physicochemical characterization of PM_2.5_, as well as the preparation of PM_2.5_ suspensions, were described in our previous studies [25,26,27]. There were 15 polycyclic aromatic hydrocarbons and 31 elements in PM_2.5_, and their concentrations are listed in Table S1.

### 2.2. Animals and Exposure Experiments

Eight-week-old male and female C57BL/6J mice were provided by Beijing Vital River Laboratory Animal Technology Co., Ltd. (Beijing, China). After one week of acclimation, male and female mice were randomly divided into the PM_2.5_-exposed and vehicle groups, 15 mice per group. Mice in the PM_2.5_-exposed group were administrated PM_2.5_ (3 mg/kg bw.) via nasal drip once every other day for 8 weeks, while mice in the vehicle control group were administered with an equal dose of the vehicle solution (blank quartz filters extraction). Urine was collected using metabolic cages in the eighth week of exposure. After exposure, blood was collected, the mice were sacrificed, and the kidneys were also collected and weighed, three of which from two groups were fixed using tissue fixation solution (*n* = 3).

### 2.3. Kidney Function Tests

Serum levels of creatinine (CRE), blood urea nitrogen (BUN), and uric acid (UA), as well as the levels of CRE and BUN in the urine, were determined using commercial kits (Nanjing Jiancheng Bioengineering Institute, Nanjing, Jiangsu, China). The estimated glomerular filtration rate (e-GFR) was calculated using the Cockcroft–Gault formula [28,29].

$$e-GFR=\frac{\left(140-age\right)\times weight\times 0.85(if\;female)}{72\times serum\;creatinine}$$

### 2.4. Measurements of ATP Content

Adenosine triphosphate (ATP) in the kidneys was determined using commercial kits (Beyotime Biotechnology, Shanghai, China).

### 2.5. Histological Analyses

Kidneys were fixed in 4% paraformaldehyde and then embedded in paraffin to prepare longitudinal kidney sections, which were stained with hematoxylin and eosin (H&E). The sections were observed using a microscope (OLYMPUS, Tokyo, Japan). Glomerulus analysis was performed to estimate the areas of the Bowman’s capsule and glomerulus using the freehand selection tool in ImageJ software. Thirty glomerular cross-sectional areas were measured in each group. The Bowman’s space was determined by subtracting the glomeruli area from the Bowman’s capsule area.

### 2.6. Enzyme-Linked Immunosorbent Assay (ELISA)

The levels of microalbumin in the urine and the levels of angiotensin II (Ang II) in the serum were measured using commercial ELISA kits obtained from Animalunion Biotechnology (Shanghai, China).

### 2.7. Quantitative RT-PCR

The extraction, reverse transcription, and storage of total RNA from the kidneys of female mice were conducted as described previously [30]. The expression of genes was determined on a qTOWER 2.2 real-time PCR system (Analytic Jena AG, Jena, Germany) using TB Premix Ex Taq II kits (TaKaRa Bio, Shiga, Kyoto, Japan). The primers used are shown in Table 2.

### 2.8. Data Analysis

Data are expressed as the mean ± standard error (SEM) and were analyzed using GraphPad Prism 8. Normal distribution was confirmed by the Shapiro–Wilk test (*p* > 0.05). Unpaired two-tailed t-tests were used to examine differences between the exposure group and the vehicle group. Differences were considered statistically significant when *p* < 0.05.

## 3. Results and Discussion

### 3.1. PM_2.5_ Exposure Causes Early Renal Injury in Female Mice

**Figure 2:**
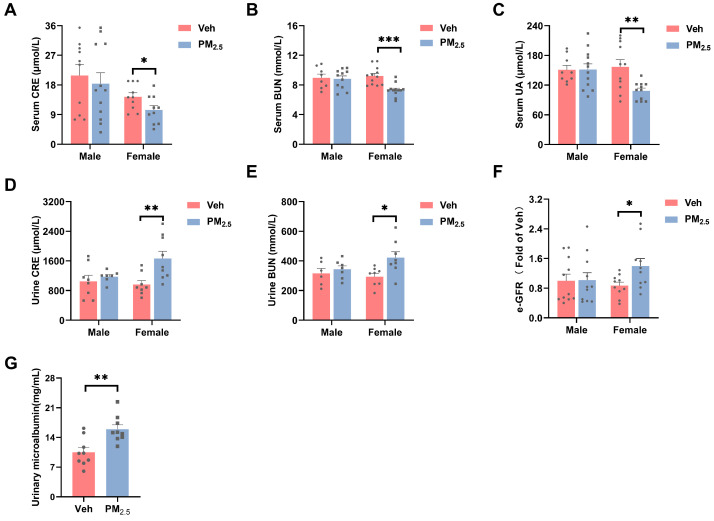
Effects of PM_2.5_ on the renal function in mice. (**A**–**C**) Serum levels of CRE, BUN, and UA in mice. (**D**,**E**) Urine levels of CRE and BUN in mice. (**F**) Estimated glomerular filtration rate (e-GFR) of kidneys in mice. (**G**) Level of urinary microalbumin in female mice. The values are expressed as the mean ± SEM (*n* ≥ 6). * *p* < 0.05, ** *p* < 0.01, *** *p* < 0.001. Veh, vehicle.

CRE, BUN, and UA are metabolites excreted mainly through the kidneys and are all important indicators for assessing renal function and glomerular filtration [31,32]. Variation in GFR is a well-known phenomenon and the natural evolution of the glomerular damage observed during chronic degenerative diseases or after exposure to nephrotoxic substances. Similarly, changes in renal function and GFR induced by PM_2.5_ exposure vary among existing studies [11,33,34,35]. In our study, the levels of serum CRE (*p* = 0.0429), BUN (*p* = 0.0002), and UA (*p* = 0.0052) were significantly lower in PM_2.5_-exposed female mice than they were in the vehicle group, whereas the levels of CRE (*p* = 0.0080) and BUN (*p* = 0.0161) in the urine were significantly elevated (Figure 2A–E), which suggests increased glomerular filtration. A significant increase in glomerular filtration levels was calculated in female mice but not in male mice (*p* = 0.0290) (Figure 2F). This indicates that female mice were more susceptible to kidney damage caused by subchronic exposure to PM_2.5_ than males.

Increased GFR implied that the kidney might be in a compensatory state, accompanied by increased glomerular capillary pressure, damaged endothelial cells, and increased capillary permeability, subsequently causing increased glomerular volume and proximal tubular load. Mehmet Kanbay et al. found that elevated GFR occurs in the early stages of kidney injury [36]. Urinary microalbumin is the most sensitive and reliable index for early diagnosis of renal function [37,38]. Importantly, the level of urinary microalbumin increased significantly in female mice exposed to PM_2.5_ (*p* = 0.0026) (Figure 2G), proving that PM_2.5_ exposure caused early kidney injury in female mice.

### 3.2. PM_2.5_ Exposure Alters Renal Pathomorphology in Female Mice

**Figure 3:**
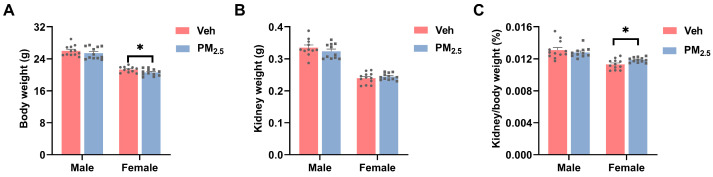
Effects of PM_2.5_ exposure on (**A**) body weight, (**B**) kidney weight, and (**C**) kidney/body weight ratio. The values are expressed as the mean ± SEM (*n* = 10–12). * *p* < 0.05. Veh, vehicle.

Function influences structure and vice versa. Following eight-week exposure to PM_2.5_, we found that female mice had a significantly decreased body weight (*p* = 0.0327), whereas the body weight of male mice did not exhibit any changes (Figure 3A). Furthermore, although both genders of mice showed no obvious alteration in kidney weight, the kidney/body weight ratio (%) of female mice showed a notable upward trend (*p* = 0.0129) (Figure 3B,C). Therefore, we supposed that the kidneys of female mice underwent compensatory enlargement following early injury. Similarly, Aurora Pérez-Gomez et al. discovered that the kidney activates hypertrophic molecular mechanisms that counteract the loss of renal function in the early stages [39]. Furthermore, early in the onset of diabetes, the kidneys enlarge due to glomerular enlargement [40].

**Figure 4:**
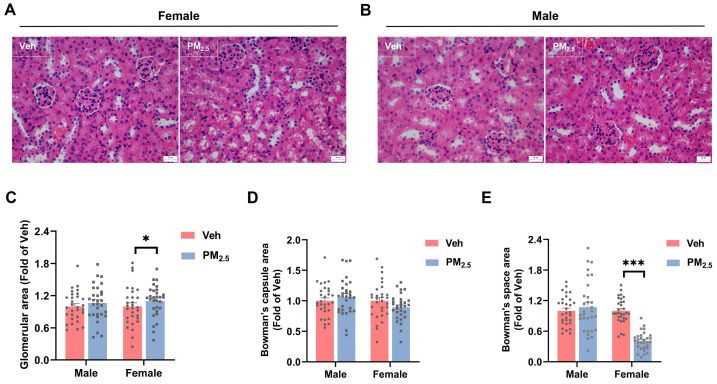
Effects of PM_2.5_ exposure on renal pathomorphology in mice. (**A**) HE staining of the kidneys in female mice (magnification: 400×), and (**B**) HE staining of the kidneys in male mice (magnification: 400×). (**C**) Glomerular area. (**D**) Bowman’s capsule area. (**E**) Bowman’s space area. The values are expressed as the mean ± SEM (*n* = 30). * *p* < 0.05, *** *p* < 0.001. Veh, vehicle.

According to previous studies, the enlargement of glomerular volume is one of the reasons for hyperfiltration. Glomerular hypertrophy has been implicated in the pathogenesis of several renal diseases, including diabetes mellitus, obesity-associated nephropathy, and focal segmental glomerulosclerosis [36,40,41,42]. The histological staining of kidney sections showed that PM_2.5_-exposed female mice exhibited enlarged glomerulus that filled the entire Bowman’s capsule, and the renal tubular cells were enlarged and tightly packed, whereas no changes were found in the kidneys of PM_2.5_-exposed male mice (Figure 4A,B). It was statistically verified that the glomerular area was significantly elevated (*p* = 0.0431) and the Bowman’s capsule area was unchanged in female mice after PM_2.5_ exposure, resulting in a remarkable decrease in the Bowman’s space area (*p <* 0.0001), whereas none of the males were changed (Figure 4C–E). Octavio Gamaliel Aztatzi-Aguilar et al. found that glomerular hypertrophy may be caused by glomerular inflammation, an imbalance of the RAS and KKS, and changes in blood pressure [11]. In addition, elevated blood glucose could also cause microvascular and podocyte damage, which in turn causes glomerular hypertrophy and kidney damage [43,44].

### 3.3. PM_2.5_ Exposure Causes Early Kidney Damage by Inducing the Imbalance of the Renin–Angiotensin System (RAS) and the Kallikrein–Kinin System (KKS)

**Figure 5:**
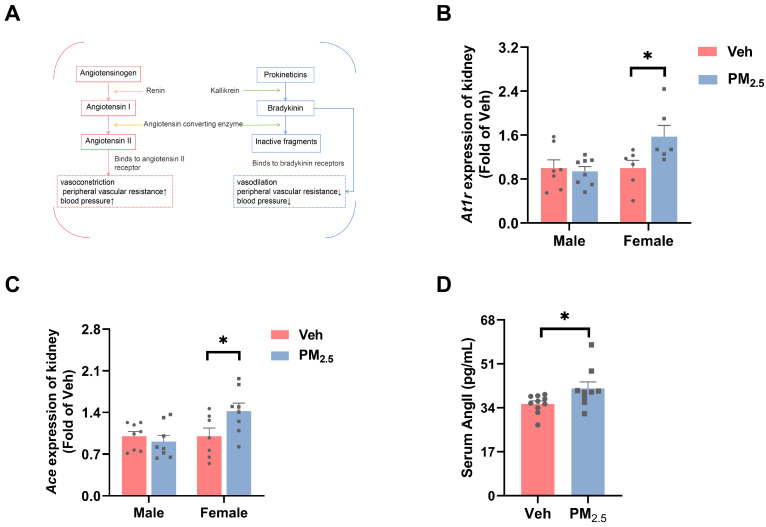
Effects of PM_2.5_ exposure on the RAS in the kidney of mice. (**A**) Interactions of the RAS and KKS. (**B**,**C**) mRNA expression of RAS-related genes. (**D**) Serum levels of angiotensin II (Ang II) in female mice. The values are expressed as the mean ± SEM (*n* ≥ 6). * *p* < 0.05. Veh, vehicle.

In addition to increased glomerular volume, an increase in glomerular capillary pressure can also result in glomerular hyperfiltration. The RAS and KKS play a crucial role in maintaining renal hemodynamics and transport function, as well as sodium and water reabsorption in distal renal units [34,35]. Increased activity and overexpression of relevant genes in the RAS and its imbalance with the KKS are among the most widely associated mechanisms of kidney disease. PM_2.5_ exposure leads to elevated inflammation and altered endocrine signaling in the lung, which in turn trigger an imbalance between the renal RAS and KKS, affects glomerular filtration, and causes kidney damage [10,11,45,46]. In our past studies, we found that PM_2.5_ exposure causes lung inflammation in mice [47]. Vasodilatation of the afferent arterioles and/or vasoconstriction of the efferent arterioles can increase the hydrostatic pressure in the glomerular capillaries, leading to glomerular hyperfiltration [36]. In the RAS, renin acts on plasma angiotensinogen, producing inactive angiotensin I (Ang I). Ang I is hydrolyzed by Ace to active Ang II, which can cause vasoconstriction of small arteries and increase blood pressure. In the KKS, Klk-1 converts kininogen to bradykinin, the latter binds to bradykinin receptors, exerting vasodilation and blood pressure-lowering effects. At the same time, Ace acts on bradykinin to convert it into inactive fragments [45,48]. These two systems are interdependent and function in regulating blood pressure and renal function (Figure 5A) [49,50,51].

Octavio Gamaliel Aztatzi-Aguilar et al. found that acute and subchronic exposure to PM_2.5_ induces the activation of the RAS and KKS [52]. However, the gender dependence of the expression of this endocrine signaling for indirect renal effects remains unclear. Consequently, we first determined the mRNA expression of angiotensin-converting enzyme (*Ace*) and angiotensin II type 1 receptor (*At1r*) in the RAS (Figure 5B,C). These genes are engaged in the endocrine pathway and angiotensin production. The results showed that the expression of *At1r* and *Ace* increased by 57.3% (*p* = 0.0438) and 42.5% (*p* = 0.0452), respectively, in PM_2.5_-exposed female mice compared with those in vehicle female mice, whereas no changes were observed in male mice. Consistent with our findings, the expression of Ace and At1r was increased in human kidney-2 (HK-2) cells exposed to PM_2.5_ and the kidneys of acutely exposed male rats [11,53]. Following this observation, we examined Ang II in the serum of female mice and found that elevated expression of Ace did increase Ang II in the serum (*p* = 0.0406) (Figure 5D), which can bind to *At1r* and cause vasoconstriction of the efferent arterioles, thus contributing to glomerular hyperfiltration [54]. On the other hand, Ang II can result in a high GFR by weakening the structural integrity of the slit diaphragm and affecting glomerular permeability [55,56].

**Figure 6:**
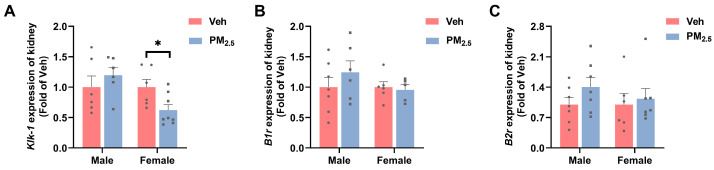
Effects of PM_2.5_ exposure on the KKS and inflammation in the kidneys of female mice. (**A**–**C**) mRNA expression of *Klk-1*, *B1r*, and *B2r*. The values are expressed as the mean ± SEM (*n* ≥ 6). * *p* < 0.05. Veh, vehicle.

Moreover, we examined the mRNA expression of kallikrein 1 (*Klk-1*), bradykinin 1 receptor (*B1r*), and bradykinin 2 receptor (*B2r*) in the KKS (Figure 6A–C), which are important mediators of vasodilation and inflammatory responses. Under pathological conditions, *B1r* expression increases in inflamed tissues [45]. The results demonstrated that the mRNA level of *Klk-1* in female mice was reduced by 37.8% (*p* = 0.0294) after PM_2.5_ exposure, and the mRNA levels of *B1r* and *B2r* were not dramatically altered. In male mice, all of them underwent non-significant changes. At the same time, we examined the mRNA expression of interleukin 6 (*Il-6)* and tumor necrosis factor-α (*Tnf-α*) (Figure S1A,B), which exhibited few alterations in these two genes, suggesting that PM_2.5_ exposure did not cause inflammation in the kidneys. Decreased *Klk-1* expression with increased *Ace* expression results in decreased bradykinin production and increased consumption, which further leads to elevated glomerular capillary blood pressure. Similarly, O. G. Aztatzi-Aguilar et al. found that PM_2.5_ exposure induces early renal injury in rats by inducing an imbalance between the RAS and the KKS [45]. Kallikrein–kinin inhibits apoptosis, inflammation, hypertrophy, and fibrosis but promotes angiogenesis and neuroregeneration in the heart, kidney, brain, and blood vessels. It is worth noting that decreased kallikrein expression can reduce this unique ability of kallikrein–kinin to repair renal tubular injury [43].

In summary, PM_2.5_ exposure may increase the glomerular capillary hydrostatic pressure by causing an imbalance of the RAS and KKS, which in turn leads to mild glomerular damage.

### 3.4. PM_2.5_ Exposure Causes Early Kidney Damage by Impacting TGF

Tubuloglomerular feedback (TGF) is one of the essential mechanisms in the self-regulation of renal blood flow and GFR, and it is highly dependent on ATP and adenosine. When macula densa cells sense an increase in sodium chloride concentration in the renal tubular fluid, it stimulates the hydrolysis of ATP to adenosine, which is then released extracellularly and acts on the A1 adenosine receptor (*A1ar*) in the afferent arterioles, activating TGF and leading to the constriction of the afferent arterioles and a lower glomerular filtration rate [57,58].

#### 3.4.1. PM_2.5_ Exposure Influences TGF by Enhancing Renal Tubule Reabsorption of Glucose

**Figure 7:**
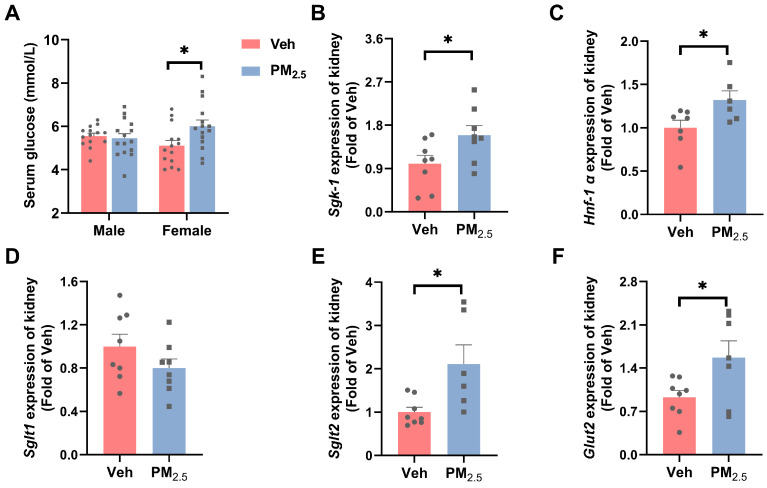
Effects of PM_2.5_ exposure on renal tubular reabsorption of glucose in female mice. (**A**) Blood glucose levels in mice. (**B**–**F**) mRNA expression of reabsorption-related genes. The values are expressed as the mean ± SEM (*n* ≥ 6). * *p* < 0.05. Veh, vehicle.

Epidemiological studies have shown that for each 1 μg/m^3^ increase in PM_2.5_, the odds of impaired fasting blood glucose increase by 10.20% in non-diabetic adolescents [59]. Toxicological studies have demonstrated that PM_2.5_ specifically affects insulin sensitivity and hepatic lipid metabolism in female mice [60]. In our work, there was a significant increase in blood glucose in female mice (*p* = 0.0240) but not in male mice (Figure 7A). Research has indicated that during the initial phases of diabetic nephropathy, there is an increase in the GFR [40]. Elevated blood glucose can increase renal blood flow and intraglomerular pressure through osmotic pressure, which increases the amount of glucose filtered through the glomerulus, thereby increasing glucose load, exposure, and reabsorption in the renal tubules.

The process of glucose reabsorption in the renal tubules is mainly dependent on sodium-dependent glucose transporters (*Sglts*) and glucose transporters (*Gluts*). Under hyperglycemia, serum- and glucocorticoid-inducible kinase 1 (*Sgk-1*) and hepatocyte nuclear factor-1α (*Hnf-1α*) can upregulate the expression of *Sglt1* and *Sglt2*, respectively, thereby increasing glucose reabsorption [61,62]. In our study, we found significantly upregulated expression of *Hnf-1α* (*p* = 0.0376), *Sgk-1* (*p* = 0.0414), *Sglt2* (*p* = 0.0167), and *Glut2* (*p* = 0.0368) in female mice following PM_2.5_ exposure, whereas no significant change was found in the expression of *Sglt1* (Figure 7B–F), but *Sglt2* plays a greater role than *Sglt1* during reabsorption [63]. These results indicated that glucose reabsorption was elevated in the renal tubules of PM_2.5_-exposed female mice. When the glomeruli are exposed to high concentrations of blood glucose, the glucose content in glomerular filtrate increases, which causes increased reabsorption of glucose from the proximal tubule, accompanied by increased reabsorption of sodium chloride in the proximal tubules, leading to a decrease in the concentration of sodium chloride in the distal tubule, as perceived by the macula densa cells; in this case, TGF was attenuated, thus leading to glomerular hyperfiltration [64,65]. SGLT2 inhibitors inhibit sodium and glucose reabsorption, leading to increased sodium in the macula densa, TGF activation, decreased glomerular hyperperfusion, high blood pressure, hyperfiltration, and recovery of renal function [66].

#### 3.4.2. PM_2.5_ Exposure Influences TGF by Inducing Renal Hypoxia and Decreased ATP Synthesis

**Figure 8:**
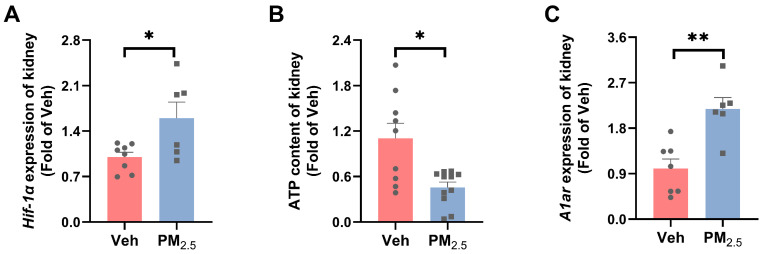
**Effects of** PM_2.5_ exposure on renal hypoxia and ATP synthesis in female mice. (**A**) mRNA expression of Hif-1α. (**B**) ATP levels. (**C**) mRNA expression of A1ar. The values are expressed as the mean ± SEM (*n* ≥ 6). * *p* < 0.05, ** *p* < 0.01. Veh, vehicle.

As explained above, the concentration of sodium chloride in the macula densa was reduced, leading to an elevation in renin release, which further increased the production of Ang II and enhanced the vasoconstrictor effects [67]. Vasoconstriction, as well as enhanced tubular reabsorption, consumes large amounts of oxygen and ATP, resulting in renal hypoxic/ischemic injury [68]. Alberto Valdés et al. proved that exposure of renal proximal tubular cells to high glucose and hypoxic conditions decreases the synthesis of ATP [69]. Kiefer W Kious et al. indicated that chronic intermittent hypoxia leads to a higher glomerular filtration rate in rats [70]. Hypoxia-inducible factor 1α (*Hif-1α*) is a key mediator that adapts cells to hypoxia [71]. In this study, the mRNA level of *Hif-1α* in the kidneys of female mice was elevated (*p* = 0.0224) (Figure 8A), indicating that the kidney might be hypoxic. Renal hypoxia can cause mitochondrial dysfunction and affect ATP synthesis. Furthermore, we verified that PM_2.5_ exposure significantly reduced the ATP content in the kidney of female mice (*p* = 0.0123) (Figure 8B), which could affect TGF and cause glomerular hyperfiltration. Notably, the mRNA levels of renal A1ar increased significantly in female mice after PM_2.5_ exposure (*p* = 0.0019) (Figure 8C). This may be a compensatory increase in the inhibition of TGF action. Therefore, we concluded that PM_2.5_ could cause mild kidney damage by affecting TGF, mainly because of enhanced renal tubule reabsorption of glucose and renal hypoxia.

## 4. Conclusions

**Figure 9:**
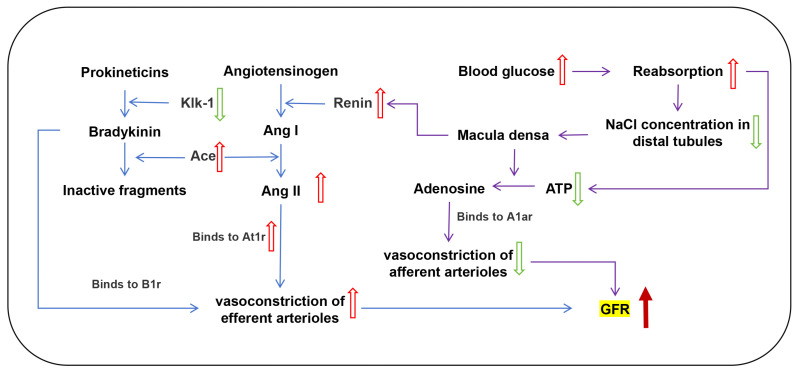
Diagram of the mechanism underlying renal injury in female mice caused by PM_2.5_ exposure. (Red and dark red arrows represent ascending. Green arrows represent descending).

In the current study, we proved that eight weeks of PM_2.5_ exposure induced early renal injury in female mice, manifested as glomerular hyperfiltration. The underlying reason for this was, on the one hand, that PM_2.5_ induced an imbalance of the RAS and KKS in female mice. On the other hand, PM_2.5_ caused elevated blood glucose in female mice, which in turn enhanced the reabsorption of glucose by the renal tubules, affected TGF, and resulted in renal hypoxia and decreased ATP (Figure 9). Our study provides valuable experimental evidence for the nephrotoxicity of PM_2.5_ and elucidates the possible mechanisms underlying the renal effects of subchronic exposure to PM_2.5_. However, as a typical outdoor air pollutant, exposure to PM_2.5_ via oropharyngeal aspiration does not fully and accurately reflect its effects on the kidneys in the real environment. Therefore, it is of interest to explore the renal effects of PM_2.5_ exposure in real environments. Additionally, due to the heterogeneity of PM_2.5_, there is a need for a deeper exploration of the effects of specific components of PM_2.5_ on kidney damage and their contribution to gender differences.

## Figures and Tables

**Table 1:** The list of abbreviations.

Full Name	Abbreviations
fine particulate matter	PM_2.5_
polycyclic aromatic hydrocarbons	PAHs
carbonaceous particles	CP
disability-adjusted life years	DALYs
end-stage renal disease	ESRD
glomerular filtration rate	GFR
creatinine	CRE
blood urea nitrogen	BUN
and uric acid	UA
Estimated glomerular filtration rate	e-GFR
adenosine triphosphate	ATP
hematoxylin and eosin	H&E
Enzyme-Linked Immunosorbent Assay	ELISA
angiotensin II	Ang II
mean ± standard error	SEM
renin–angiotensin system	RAS
kallikrein–kinin system	KKS
angiotensin I	Ang I
angiotensin-converting enzyme	*Ace*
angiotensin II type 1 receptor	*At1r*
Human Kidney-2	HK-2
kallikrein 1	*Klk-1*
bradykinin 1 receptor	*B1r*
bradykinin 2 receptor	*B2r*
interleukin 6	*Il-6*
tumor necrosis factor-α	*Tnf-* *α*
sodium-dependent glucose transporters	*Sglts*
glucose transporters	*Gluts*
serum- and glucocorticoid-inducible kinase 1	*Sgk-1*
hepatocyte nuclear factor -1α	*Hnf-1α*
tubuloglomerular feedback	TGF
A1 adenosine receptor	*A1ar*
Hypoxia-inducible factor 1α	*Hif-1α*

**Table 2:** Primer sequences of the genes used in quantitative RT-PCR.

Gene	Primer Sequence (5′-3′)
*Ace*	*F: CTCCGCTCTTGATGCTGTC*
	*R: TTCTCCTCCGTGATGTTGGT*
*At1r*	*F: ATGTTTCTTGGTGGCTTGGTT*
	*R: CAGCAGCGTCTGATGATGAG*
*Klk-1*	*F: CAATGTGGGGGTATCCTGCTG*
	*R: GGGTATTCATATTTGACGGGTGT*
*B1r*	*F: TCCTTCTGCGTTCCGTCAA*
	*R: TTCAACTCCACCATCCTTACAA*
*B2r*	*F: AGGTGCTGAGGAACAACGA*
	*R: AGGAAGGTGCTGATCTGGAA*
*Il-6*	*F: TGATGGATGCTACCAAACTGGA*
	*R: TGTGACTCCAGCTTATCTCTTGG*
*Tnf-* *α*	*F: CCACGCTCTTCTGTCTACTGA*
	*R: GTTTGTGAGTGTGAGGGTCTG*
*Sgk-1*	*F: GGCACAAGGCAGAAGAAGTATT*
	*R: GGTCTGGAATGAGAAGTGAAGG*
*Hnf-1α*	*F: GACCTGACCGAGTTGCCTAAT*
	*R: CCGGCTCTTTCAGAATGGGT*
*Sglt1*	*F: CTCTTCGTCATCAGCGTCATC*
	*R: TCCTCCTCCTCCTTAGTCATCT*
*Sglt2*	*F: TCAGAACCAATAGAGGCACAGT*
	*R: CGGACAGGTAGAGGCGAATA*
*Glut2*	*F: GTCACACCAGCATACACAACA*
	*R: ACTTCGTCCAGCAATGATGAG*
*A1ar*	*F: ATCCTGGCTCTGCTTGCTATT*
	*R: GGCTTGTTCCACCTCACTCA*
*Hif-1* *α*	*F: ACCTTCATCGGAAACTCCAAAG*
	*R: CTCTTAGGCTGGGAAAAGTTAGG*
*Gapdh*	*F: AGAAGGTGGTGAAGCAGGCATC*
	*R: GATGGACTTCGGGAACGGACAG*
*β-Actin*	*F: GCTTCTTTGCAGCTCCTTCGT*
	*R: ATATCGTCATCCATGGCGAAC*

## Data Availability

Data are contained within the article.

## Supplementary Materials

The following are available online at https://www.mdpi.com/article/10.3390/toxics12120878/s1, Figure S1. Effects of PM_2.5_ exposure inflammation in the kidney of mice. (A,B) The mRNA expression of inflammatory factor, Il-6 and Tnf-α. The values are expressed as the mean ± SEM (n ≥ 6). Veh, vehicle, Table S1. The contents of elements and polycyclic aromatic hydrocarbons in PM_2.5_ samples.

## References

[1] Yan R., Ma D., Liu Y., Wang R., Fan L., Yan Q., Chen C., Wang W., Ren Z., Ku T. (2024). Developmental Toxicity of Fine Particulate Matter: Multifaceted Exploration from Epidemiological and Laboratory Perspectives. Toxics.

[2] Jiang Y., Peng Y., Yang X., Yu J., Yu F., Yuan J., Zha Y. (2023). PM_2.5_ Exposure Aggravates Kidney Damage by Facilitating the Lipid Metabolism Disorder in Diabetic Mice. PeerJ.

[3] Sang S., Chu C., Zhang T., Chen H., Yang X. (2022). The Global Burden of Disease Attributable to Ambient Fine Particulate Matter in 204 Countries and Territories, 1990–2019: A Systematic Analysis of the Global Burden of Disease Study 2019. Ecotoxicol. Environ. Saf..

[4] Chaudhary E., George F., Saji A., Dey S., Ghosh S., Thomas T., Kurpad A.V., Sharma S., Singh N., Agarwal S. (2023). Cumulative Effect of PM_2.5_ Components Is Larger than the Effect of PM_2.5_ Mass on Child Health in India. Nat. Commun..

[5] Bandyopadhyay A. (2016). Neurological Disorders from Ambient (Urban) Air Pollution Emphasizing UFPM and PM_2.5_. Curr. Pollut. Rep..

[6] Yan R., Ji S., Ku T., Sang N. (2024). Cross-Omics Analyses Reveal the Effects of Ambient PM_2.5_ Exposure on Hepatic Metabolism in Female Mice. Toxics.

[7] Lin S.-Y., Ju S.-W., Lin C.L., Hsu W.-H., Lin C.-C., Ting I.-W., Kao C.-H. (2020). Air Pollutants and Subsequent Risk of Chronic Kidney Disease and End-Stage Renal Disease: A Population-Based Cohort Study. Environ. Pollut..

[8] An Y., Liu Z.-H. (2021). Air Pollution and Kidney Diseases: PM_2.5_ as an Emerging Culprit. Contrib. Nephrol..

[9] Seltenrich N. (2016). PM_2.5_ and Kidney Function: Long-Term Exposures May Lead to Modest Declines. Environ. Health Perspect..

[10] Xu W., Wang S., Jiang L., Sun X., Wang N., Liu X., Yao X., Qiu T., Zhang C., Li J. (2022). The Influence of PM_2.5_ Exposure on Kidney Diseases. Hum. Exp. Toxicol..

[11] Aztatzi-Aguilar O.G., Pardo-Osorio G.A., Uribe-Ramírez M., Narváez-Morales J., De Vizcaya-Ruiz A., Barbier O.C. (2021). Acute Kidney Damage by PM_2.5_ Exposure in a Rat Model. Environ. Toxicol. Pharmacol..

[12] Lin C.-H., Wan C., Liu W.-S., Wang H.-H. (2021). PM_2.5_ Induces Early Epithelial Mesenchymal Transition in Human Proximal Tubular Epithelial Cells through Activation of IL-6/STAT3 Pathway. Int. J. Mol. Sci..

[13] Pei H., Dai X., He Z., Tang Z., Zhu Y., Du R. (2024). PM_2.5_ Exposure Promotes the Progression of Acute Kidney Injury by Activating NLRP3-Mediated Macrophage Inflammatory Response. Ecotoxicol. Environ. Saf..

[14] Lu X., Chen K., Zeng J., Ren H., Zeng C. (2014). Abstract 281: Long-Term Exposure of PM_2.5_ Causes Hypertension by Impaired Renal D1 Receptor Mediated Sodium Excretion via Up-Regulation of GRK4 Expression in SD Rats. Hypertension.

[15] Lash L.H. (2019). Environmental and Genetic Factors Influencing Kidney Toxicity. Semin. Nephrol..

[16] Kelly D.M., Anders H.-J., Bello A.K., Choukroun G., Coppo R., Dreyer G., Eckardt K.-U., Johnson D.W., Jha V., Harris D.C.H. (2021). International Society of Nephrology Global Kidney Health Atlas: Structures, Organization, and Services for the Management of Kidney Failure in Western Europe. Kidney Int. Suppl..

[17] Harnois T., Brishoual S., Vincent-Tassin E., Paris I., Bourmeyster N., Hadjadj S. (2012). O46 Les Fluctuations Du Glucose Induisent Une Activation de La Fibrose Rénale Par Des Voies de Signalisation P38-MAP Kinase et Rho/Rock. Diabetes Metab..

[18] Cui Y., Fang J., Guo H., Cui H., Deng J., Yu S., Gou L., Wang F., Ma X., Ren Z. (2022). Notch3-Mediated mTOR Signaling Pathway Is Involved in High Glucose-Induced Autophagy in Bovine Kidney Epithelial Cells. Molecules.

[19] Ji S., Guo Y., Yan W., Wei F., Ding J., Hong W., Wu X., Ku T., Yue H., Sang N. (2024). PM_2.5_ Exposure Contributes to Anxiety and Depression-like Behaviors via Phenyl-Containing Compounds Interfering with Dopamine Receptor. Proc. Natl. Acad. Sci. USA.

[20] Takasato M., Er P.X., Chiu H.S., Maier B., Baillie G.J., Ferguson C., Parton R.G., Wolvetang E.J., Roost M.S., Chuva de Sousa Lopes S.M. (2015). Kidney Organoids from Human iPS Cells Contain Multiple Lineages and Model Human Nephrogenesis. Nature.

[21] Gupta N., Morizane R. (2022). Kidney Development to Kidney Organoids and Back Again. Semin. Cell Dev. Biol..

[22] Tekguc M., Gaal R.C.V., Uzel S.G.M., Gupta N., Riella L.V., Lewis J.A., Morizane R. (2022). Kidney Organoids: A Pioneering Model for Kidney Diseases. Transl. Res..

[23] Xin Y., Liu Y., Liu L., Wang X., Wang D., Song Y., Shen L., Liu Y., Liu Y., Peng Y. (2024). Dynamic Changes in the Real-Time Glomerular Filtration Rate and Kidney Injury Markers in Different Acute Kidney Injury Models. J. Transl. Med..

[24] Sancho-Martínez S.M., Blanco-Gozalo V., Quiros Y., Prieto-García L., Montero-Gómez M.J., Docherty N.G., Martínez-Salgado C., Morales A.I., López-Novoa J.M., López-Hernández F.J. (2020). Impaired Tubular Reabsorption Is the Main Mechanism Explaining Increases in Urinary NGAL Excretion Following Acute Kidney Injury in Rats. Toxicol. Sci..

[25] Ku T., Li B., Gao R., Zhang Y., Yan W., Ji X., Li G., Sang N. (2017). NF-κB-Regulated microRNA-574-5p Underlies Synaptic and Cognitive Impairment in Response to Atmospheric PM_2.5_ Aspiration. Part. Fibre Toxicol..

[26] Xing Q., Wu M., Chen R., Liang G., Duan H., Li S., Wang Y., Wang L., An C., Qin G. (2021). Comparative Studies on Regional Variations in PM_2.5_ in the Induction of Myocardial Hypertrophy in Mice. Sci. Total. Environ..

[27] Hou Y., Yan W., Guo L., Li G., Sang N. (2023). Prenatal PM_2.5_ Exposure Impairs Spatial Learning and Memory in Male Mice Offspring: From Transcriptional Regulation to Neuronal Morphogenesis. Part. Fibre Toxicol..

[28] Rivera-Caravaca J.M., Ruiz-Nodar J.M., Tello-Montoliu A., Esteve-Pastor M.A., Quintana-Giner M., Véliz-Martínez A., Orenes-Piñero E., Romero-Aniorte A.I., Vicente-Ibarra N., Pernias-Escrig V. (2018). Disparities in the Estimation of Glomerular Filtration Rate According to Cockcroft-Gault, Modification of Diet in Renal Disease-4, and Chronic Kidney Disease Epidemiology Collaboration Equations and Relation With Outcomes in Patients With Acute Coronary Syndrome. J. Am. Heart Assoc..

[29] Kumar B.V., Mohan T. (2017). Retrospective Comparison of Estimated GFR Using 2006 MDRD, 2009 CKD-EPI and Cockcroft-Gault with 24 Hour Urine Creatinine Clearance. J. Clin Diagn. Res..

[30] Guo Y., Ji S., Rong S., Hong W., Ding J., Yan W., Qin G., Li G., Sang N. (2024). Screening Organic Components and Toxicogenic Structures from Regional Fine Particulate Matters Responsible for Myocardial Fibrosis in Male Mice. Environ. Sci. Technol..

[31] Shekoufeh A., Hassanali A., Nazanin S.J., Mohammad Aref B., Jamileh S., Amirashkan M., Hossein K.J. (2023). Effect of High Doses of Salep Aqueous Extract on Serum Levels of Urea Nitrogen, Creatinine, Uric Acid, and Kidney Histopathological Changes in Adult Male Wistar Rats. Arch. Razi. Inst..

[32] Mirsharif E.S., Heidary F., Vaez Mahdavi M.R., Gharebaghi R., Pourfarzam S., Ghazanfari T. (2018). Sulfur Mustard-Induced Changes in Blood Urea Nitrogen, Uric Acid and Creatinine Levels of Civilian Victims, and Their Correlation with Spirometric Values. Iran. J. Public. Health.

[33] Paoin K., Ueda K., Vathesatogkit P., Ingviya T., Buya S., Dejchanchaiwong R., Phosri A., Seposo X.T., Kitiyakara C., Thongmung N. (2022). Long-Term Air Pollution Exposure and Decreased Kidney Function: A Longitudinal Cohort Study in Bangkok Metropolitan Region, Thailand from 2002 to 2012. Chemosphere.

[34] Wu Y.-H., Wu C.-D., Chung M.-C., Chen C.-H., Wu L.-Y., Chung C.-J., Hsu H.-T. (2022). Long-Term Exposure to Fine Particulate Matter and the Deterioration of Estimated Glomerular Filtration Rate: A Cohort Study in Patients With Pre-End-Stage Renal Disease. Front. Public Health.

[35] Nolin T.D., Himmelfarb J. (2010). Mechanisms of Drug-Induced Nephrotoxicity. Adverse Drug Reactions.

[36] Kanbay M., Copur S., Bakir C.N., Covic A., Ortiz A., Tuttle K.R. (2024). Glomerular Hyperfiltration as a Therapeutic Target for CKD. Nephrol Dial Transpl..

[37] Oz-Sig O., Kara O., Erdogan H. (2020). Microalbuminuria and Serum Cystatin C in Prediction of Early-Renal Insufficiency in Children with Obesity. Indian. J. Pediatr..

[38] Chiarelli F., Verrotti A., Morgese G. (1995). Glomerular Hyperfiltration Increases the Risk of Developing Microalbuminuria in Diabetic Children. Pediatr. Nephrol..

[39] Pérez-Gomez A., Diaz-Tocados J.M., Coral J.D.D., Enriquez M.C., García-Carrasco A., Bardaji A.M., Revilla J.M.V. (2023). #5799 Compensatory Hypertrophy of The Kidney Contributes to Loss of Klotho Kidney Through Pakt Signaling. Nephrol. Dial. Transplant..

[40] Yang Y., Xu G. (2022). Update on Pathogenesis of Glomerular Hyperfiltration in Early Diabetic Kidney Disease. Front. Endocrinol..

[41] Kataoka H., Mochizuki T., Nitta K. (2018). Large Renal Corpuscle: Clinical Significance of Evaluation of the Largest Renal Corpuscle in Kidney Biopsy Specimens. Contrib. Nephrol..

[42] Toyota E., Ogasawara Y., Fujimoto K., Kajita T., Shigeto F., Asano T., Watanabe N., Kajiya F. (2004). Global Heterogeneity of Glomerular Volume Distribution in Early Diabetic Nephropathy. Kidney Int..

[43] Stackhouse S., Miller P.L., Park S.K., Meyer T.W. (1990). Reversal of Glomerular Hyperfiltration and Renal Hypertrophy by Blood Glucose Normalization in Diabetic Rats. Diabetes.

[44] Daehn I.S. (2018). Glomerular Endothelial Cell Stress and Cross-Talk With Podocytes in Early [Corrected] Diabetic Kidney Disease. Front. Med..

[45] Aztatzi-Aguilar O.G., Uribe-Ramírez M., Narváez-Morales J., De Vizcaya-Ruiz A., Barbier O. (2016). Early Kidney Damage Induced by Subchronic Exposure to PM_2.5_ in Rats. Part. Fibre Toxicol..

[46] Lin C.-I., Tsai C.-H., Sun Y.-L., Hsieh W.-Y., Lin Y.-C., Chen C.-Y., Lin C.-S. (2018). Instillation of Particulate Matter 2.5 Induced Acute Lung Injury and Attenuated the Injury Recovery in ACE2 Knockout Mice. Int. J. Biol. Sci..

[47] Ji X., Yue H., Ku T., Zhang Y., Yun Y., Li G., Sang N. (2019). Histone Modification in the Lung Injury and Recovery of Mice in Response to PM_2.5_ Exposure. Chemosphere.

[48] da Costa P.L.N., Wynne D., Fifis T., Nguyen L., Perini M., Christophi C. (2018). The Kallikrein-Kinin System Modulates the Progression of Colorectal Liver Metastases in a Mouse Model. BMC Cancer.

[49] Koumallos N., Sigala E., Milas T., Baikoussis N.G., Aragiannis D., Sideris S., Tsioufis K. (2023). Angiotensin Regulation of Vascular Homeostasis: Exploring the Role of ROS and RAS Blockers. Int. J. Mol. Sci..

[50] Regoli D., Gobeil F. (2015). Critical Insights into the Beneficial and Protective Actions of the Kallikrein-Kinin System. Vascul. Pharmacol..

[51] Hornig B., Kohler C., Drexler H. (1997). Role of Bradykinin in Mediating Vascular Effects of Angiotensin-Converting Enzyme Inhibitors in Humans. Circulation.

[52] Aztatzi-Aguilar O.G., Uribe-Ramírez M., Arias-Montaño J.A., Barbier O., De Vizcaya-Ruiz A. (2015). Acute and Subchronic Exposure to Air Particulate Matter Induces Expression of Angiotensin and Bradykinin-Related Genes in the Lungs and Heart: Angiotensin-II Type-I Receptor as a Molecular Target of Particulate Matter Exposure. Part. Fibre Toxicol..

[53] Kang E., Yim H.E., Nam Y.J., Jeong S.H., Kim J.-A., Lee J.-H., Son M.H., Yoo K.H. (2022). Exposure to Airborne Particulate Matter Induces Renal Tubular Cell Injury in Vitro: The Role of Vitamin D Signaling and Renin-Angiotensin System. Heliyon.

[54] Pahlitzsch T., Liu Z.Z., Al-Masri A., Braun D., Dietze S., Persson P.B., Schunck W.-H., Blum M., Kupsch E., Ludwig M. (2018). Hypoxia-Reoxygenation Enhances Murine Afferent Arteriolar Vasoconstriction by Angiotensin II. Am. J. Physiol. Renal. Physiol..

[55] Königshausen E., Zierhut U.M., Ruetze M., Potthoff S.A., Stegbauer J., Woznowski M., Quack I., Rump L.C., Sellin L. (2016). Angiotensin II Increases Glomerular Permeability by β-Arrestin Mediated Nephrin Endocytosis. Sci. Rep..

[56] Axelsson J., Rippe A., Oberg C.M., Rippe B. (2012). Rapid, Dynamic Changes in Glomerular Permeability to Macromolecules during Systemic Angiotensin II (ANG II) Infusion in Rats. Am. J. Physiol. Renal. Physiol..

[57] Castrop H. (2007). Mediators of Tubuloglomerular Feedback Regulation of Glomerular Filtration: ATP and Adenosine. Acta Physiol..

[58] Osswald H., Mühlbauer B., Vallon V. (2008). Adenosine and Tubuloglomerular Feedback. Blood Purif..

[59] Yu W., Sulistyoningrum D.C., Gasevic D., Xu R., Julia M., Murni I.K., Chen Z., Lu P., Guo Y., Li S. (2020). Long-Term Exposure to PM_2.5_ and Fasting Plasma Glucose in Non-Diabetic Adolescents in Yogyakarta, Indonesia. Environ. Pollut..

[60] Li R., Sun Q., Lam S.M., Chen R., Zhu J., Gu W., Zhang L., Tian H., Zhang K., Chen L.-C. (2020). Sex-Dependent Effects of Ambient PM_2.5_ Pollution on Insulin Sensitivity and Hepatic Lipid Metabolism in Mice. Part. Fibre Toxicol..

[61] Lang F., Görlach A., Vallon V. (2009). Targeting SGK1 in Diabetes. Expert Opin. Ther. Targets.

[62] Freitas H.S., Anhê G.F., Melo K.F.S., Okamoto M.M., Oliveira-Souza M., Bordin S., Machado U.F. (2008). Na(+) -Glucose Transporter-2 Messenger Ribonucleic Acid Expression in Kidney of Diabetic Rats Correlates with Glycemic Levels: Involvement of Hepatocyte Nuclear Factor-1alpha Expression and Activity. Endocrinology.

[63] Santer R., Calado J. (2010). Familial Renal Glucosuria and SGLT2: From a Mendelian Trait to a Therapeutic Target. Clin. J. Am. Soc. Nephrol..

[64] Persson P., Hansell P., Palm F. (2010). Tubular Reabsorption and Diabetes-Induced Glomerular Hyperfiltration. Acta Physiol..

[65] Vallon V., Thomson S.C. (2020). The Tubular Hypothesis of Nephron Filtration and Diabetic Kidney Disease. Nat. Rev. Nephrol.

[66] Gonzalez D.E., Foresto R.D., Ribeiro A.B. (2020). SGLT-2 Inhibitors in Diabetes: A Focus on Renoprotection. Rev. Assoc. Med. Bras..

[67] Schnermann J. (1998). Juxtaglomerular Cell Complex in the Regulation of Renal Salt Excretion. Am. J. Physiol..

[68] Vallon V. (2011). The Proximal Tubule in the Pathophysiology of the Diabetic Kidney. Am. J. Physiol. Regul. Integr. Comp. Physiol..

[69] Valdés A., Castro-Puyana M., García-Pastor C., Lucio-Cazaña F.J., Marina M.L. (2020). Time-Series Proteomic Study of the Response of HK-2 Cells to Hyperglycemic, Hypoxic Diabetic-like Milieu. PLoS ONE.

[70] Kious K.W., Savage K.A., Twohey S.C.E., Highum A.F., Philipose A., Díaz H.S., Del Rio R., Lang J.A., Clayton S.C., Marcus N.J. (2023). Chronic Intermittent Hypoxia Promotes Glomerular Hyperfiltration and Potentiates Hypoxia-Evoked Decreases in Renal Perfusion and PO_2_. Front. Physiol..

[71] Packer M. (2021). Mechanisms Leading to Differential Hypoxia-Inducible Factor Signaling in the Diabetic Kidney: Modulation by SGLT2 Inhibitors and Hypoxia Mimetics. Am. J. Kidney Dis..

