# Supplementary material for: PM2.5 Exposure Induces Glomerular Hyperfiltration in Mice in a Gender-Dependent Manner
Source: Toxics. 2024 Dec 1;12(12):878. doi: 10.3390/toxics12120878 (PMC11679005; doi:10.3390/toxics12120878)
Supplement: Supplementary file 1 [file toxics-12-00878-s001.zip › toxics-3260213-supplementary.pdf]

**Table S1.** The contents of elements and polycyclic aromatic hydrocarbons in $PM_{2.5}$ samples[1].

| Elements ($ng/m^3$) | | Polycyclic aromatic hydrocarbon (PAHs) ($ng/m^3$) | |
|---|---|---|---|
| Ca | 1397.85 | Benzo(b)fluoranthene (BbFA) | 16.70 |
| Fe | 1197.20 | Fluoranthene (FA) | 14.58 |
| K | 1066.67 | Chrysene (CHR) | 12.20 |
| Al | 735.48 | Benzo(a)anthracene (BaA) | 11.66 |
| Zn | 323.87 | Pyrene (PY) | 11.32 |
| Cu | 194.62 | Benzo(a)pyrene (BaP) | 9.76 |
| Pb | 97.94 | Indeno(1,2,3-cd)pyrene (IP) | 8.92 |
| Mn | 93.76 | Benzo(g,h,i)perylene (BghiP) | 8.59 |
| Mg | 43.01 | Phenanthrene (PHE) | 5.66 |
| W | 27.31 | Benzo(k)gluoranthene (BkFA) | 4.07 |
| Bi | 15.85 | Dibebz(a.h)anthracene (DBahA) | 1.71 |
| Cr | 11.83 | Fluorene (FL) | 0.54 |
| As | 5.59 | Anthracene (AN) | 0.49 |
| Sn | 4.84 | Naphthalene (NA) | 0.42 |
| Ni | 4.30 | Acenaphthylene (ACL) | 0.26 |
| Rb | 3.87 | | |
| Mo | 3.87 | | |
| Li | 2.56 | | |
| Sb | 1.42 | | |
| V | 1.38 | | |
| Ce | 1.10 | | |
| Tl | 1.07 | | |
| Co | 0.75 | | |
| Cd | 0.62 | | |
| La | 0.54 | | |
| Cs | 0.51 | | |
| Y | 0.28 | | |
| Th | 0.16 | | |
| U | 0.16 | | |
| Sm | 0.06 | | |
| Be | 0.04 | | |

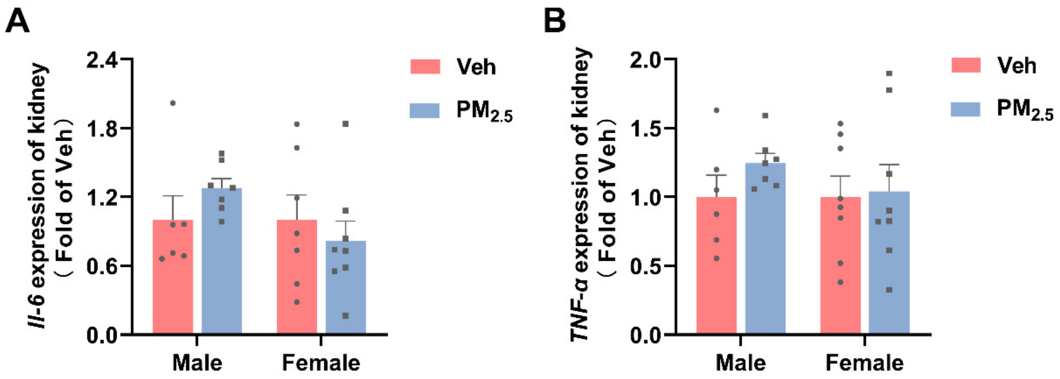


**Figure S1.** Effects of $PM_{2.5}$ exposure inflammation in the kidney of mice. (A-B) The mRNA expression of inflammatory factor, Il-6 and Tnf-$\alpha$. The values are expressed as the mean ± SEM ($n \geq 6$). Veh, vehicle.

**References**

1. Hou, Y.; Yan, W.; Guo, L.; Li, G.; Sang, N. Prenatal $PM_{2.5}$ Exposure Impairs Spatial Learning and Memory in Male Mice Offspring: From Transcriptional Regulation to Neuronal Morphogenesis. *Part. Fibre. Toxicol.* **2023**, *20*, 13.
